# Stochastic atmospheric assistance and the use of emergency staging sites by migrants

**DOI:** 10.1098/rspb.2009.2112

**Published:** 2010-01-13

**Authors:** Judy Shamoun-Baranes, Jutta Leyrer, Emiel van Loon, Pierrick Bocher, Frédéric Robin, Francis Meunier, Theunis Piersma

**Affiliations:** 1 Computational Geo-ecology, Institute for Biodiversity and Ecosystem Dynamics IBED, University of Amsterdam, Nieuwe Achtergracht 166, 1018 WV Amsterdam, The Netherlands; 2 Department of Marine Ecology, Royal Netherlands Institute for Sea Research NIOZ, PO Box 59, 1790 AB Den Burg, Texel, The Netherlands; 3 Animal Ecology Group, Centre for Ecological and Evolutionary Studies CEES, University of Groningen, PO Box 14, 7900 AA Haren, The Netherlands; 4 Marine Ecology, Laboratory of Coastal Environment and Societies UMR 6250 LIENSs CNRS-University of La Rochelle, 2 rue Olympe de Gouges, La Rochelle 17000, France; 5 Ligue pour la Protection des Oiseaux LPO, La Corderie Royale, BP 90263, 17305 Rochefort Cedex, France

**Keywords:** *Calidris canutus canutus*, conservation, migration, modelling, stopover, wind assistance

## Abstract

Numerous animals move vast distances through media with stochastic dynamic properties. Avian migrants must cope with variable wind speeds and directions *en route*, which potentially jeopardize fine-tuned migration routes and itineraries. We show how unpredictable winds affect flight times and the use of an intermediate staging site by red knots (*Calidris canutus canutus*) migrating from west Africa to the central north Siberian breeding areas via the German Wadden Sea. A dynamic migration model incorporating wind conditions during flight shows that flight durations between Mauritania and the Wadden Sea vary between 2 and 8 days. The number of birds counted at the only known intermediate staging site on the French Atlantic coast was strongly positively correlated with simulated flight times. In addition, particularly light-weight birds occurred at this location. These independent results support the idea that stochastic wind conditions are the main driver of the use of this intermediate stopover site as an emergency staging area. Because of the ubiquity of stochastically varying media, we expect such emergency habitats to exist in many other migratory systems, both airborne and oceanic. Our model provides a tool to quantify the effect of winds and currents *en route*.

## 1. Introduction

Migratory animals spend different parts of their lives in widely separated and ecologically distinct locations. Their migratory movements can be very long, energetically costly and often take place in stochastically dynamic conditions (Dingle 1996; Liechti 2006; Chapman *et al*. 2008; Gill *et al*. 2009; Sale & Luschi 2009). As a consequence, animals must store enough fuel to travel, need to stop in suitable habitats to refuel or rest, and may need to make use of environmental assistance (e.g. wind or currents) in order to migrate successfully. In birds, migration can comprise up to 50 per cent of the annual energy budget (Drent & Piersma 1990). As wind speeds are within the same order of magnitude as bird flight speeds, wind can have a strong impact on the cost and speed of migration (Liechti & Bruderer 1998; Shamoun-Baranes *et al*. 2003).

**Figure 1.**
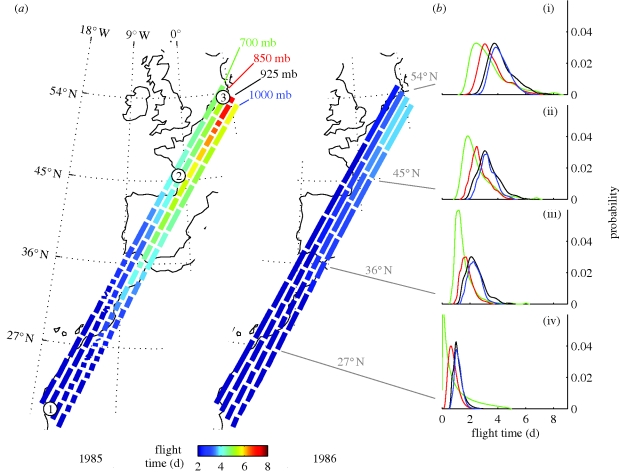
Simulated spring knot migration along the great circle route (a straight line in the chosen map projection) from Banc d'Arguin, Mauritania (circled 1), through their potential staging site along the French Atlantic Coast at (circled 2) to their main stopover in the German Wadden Sea (circled 3). (*a*) Flight trajectories for 1985 (left) and 1986 (right), with 5 May as the starting date. Each segment marks a 6 h time interval. (*b*) The probability density functions of cumulative flight at latitudes (i) up to 54° N, (ii) up to 45° N, (iii) up to 36° N and (iv) up to 27° N, over all years (1979–2007) and all starting dates (1–10 May) per pressure level.

A well-studied example of a long-distance migrant is the red knot (*Calidris canutus*). The Afro-Siberian nominate subspecies *C. c. canutus* migrates north in two non-stop flights of approximately 4400 km each, from the Mauritanian wintering grounds via the German Wadden Sea (figure 1*a*) to the Siberian breeding grounds in only four weeks (Piersma *et al*. 1992; van de Kam *et al*. 2004). Although red knots and other wader species worldwide routinely make such long non-stop flights (Pennycuick & Battley 2003; van de Kam *et al*. 2004; Piersma *et al*. 2005; Gill *et al*. 2009), individuals may deplete energy stores before reaching their final destination owing to unfavourable wind conditions during flight (Piersma & van de Sant 1992; van de Kam *et al*. 2004). Mauritania is within the trade wind zone where headwinds prevail during spring migration. Previous studies failed to find correlations between surface winds and departure intensity (Piersma *et al*. 1990; Piersma & van de Sant 1992); therefore, red knots embarking from Mauritania cannot optimize departure dates on the basis of surface winds. In addition, red knots depart from Mauritania with such low body mass (Dick *et al*. 1987; Piersma *et al*. 1992) that a successful migration from Mauritania to the Wadden Sea seems unlikely without wind support.

Annually, highly variable numbers of red knots use stopover sites at the French Atlantic coastal wetlands. In some years over 50 000 individuals use these sites, which is about 25 per cent of the migratory population, whereas in other years these sites are skipped (Stroud *et al*. 2004; van de Kam *et al*. 2004; Leyrer *et al*. in press). Owing to the specialized diet of red knots, suitable refuelling sites are discretely distributed along the migratory route (Piersma 2007); thus, red knots must fly relatively large distances non-stop with sufficient energy stores before reaching the next suitable refuelling site. However, increasing energy stores increases transport costs and reduces flight manoeuvrability (Kvist *et al*. 2001; van den Hout *et al*. 2010), whereas increasing stopover frequency or duration increases migration time, which in turn can also have a negative impact on survival and individual fitness (e.g. Newton 2008).

Migration theory predicts that birds minimizing time during migration should store enough fuel in advance and save time by skipping sites with low or unpredictable food supply (Alerstam & Hedenström 1998). Thus birds trying to minimize the total amount of time spent on migration would benefit from storing extra fuel at high-quality stopover sites and bypassing sites with lower food quality or quantity. Evidence for skipping has been put forward by a few theoretical and empirical studies (Gudmundsson *et al*. 1991; Weber *et al*. 1994; Beekman *et al*. 2002). These studies focus on the importance of food quality or availability and not the potential influence of wind on stopover site usage.

Wind conditions can strongly influence the cost and speed of migration (Liechti & Bruderer 1998; Shamoun-Baranes *et al*. 2003), as well as stopover duration and take-off decisions (Åkesson & Hedenström 2000; Weber & Hedenström 2000; Åkesson *et al*. 2002; Schaub *et al*. 2004). Here we propose that irregular site use is an emergency stopover strategy of birds running out of fuel when winds are unfavourable during flight. We introduce the concept that staging site use is a function of stochastic wind conditions. Using a dynamic model, we studied the impact on flight times of winds *en route* at different flight altitudes, migratory start dates and years. Under increasingly unfavourable wind conditions *en route*, resulting in longer flight times, we expected that more birds would use the emergency staging site in France. To test this we compared modelled flight times to observed site use on the French Atlantic coast (figure 1*a*).

## 2. Material and methods

Spring migration of red knots *C. c. canutus* from their main wintering area on Banc d'Arguin, Mauritania (19°53′ N, 16°17′ W), to their staging area in the German Wadden Sea (54°01′ N, 8°48′ E; figure 1*a*) was simulated based on current knowledge of the migration system of this subspecies (Piersma *et al*. 1990, 1992; Piersma 2007). The model framework provides a way to simultaneously explore the impact of daily, annual and altitudinal wind variability on flight time along a pre-defined migration route.

### (a) Model description

A dynamic model was designed to simulate red knot spring migration using a deterministic one-dimensional ordinary differential equations model, implemented by a fixed 6 h time-step forward integration scheme. Six-hourly *u* and *v* wind components with a 2.5° × 2.5° spatial resolution were extracted for four standard pressure levels (1000, 925, 850, 700 mb; corresponding to 111, 766, 1457 and 3012 m above mean sea level, respectively), at standard atmospheric conditions (Anonymous 1976) from the NCEP-NCAR reanalysis dataset (Kalnay *et al*. 1996). In this model, birds moved along a great circle trajectory between the wintering and main stopover site. Air speed of the birds was kept constant (16 m s^−1^; Noer 1979; Gudmundsson 1994; Kvist *et al*. 2001); therefore flight time can be considered an indirect measure of flight cost by calculating mechanical power output based on aerodynamic theory (Pennycuick 2008). Ground speed was calculated at each time-step based on wind speed and direction, track heading and air speed (Shamoun-Baranes *et al*. 2007) at that time and location. Resulting flight times are a direct measure of wind assistance; lower flight times reflect stronger wind assistance. Winds along the flight trajectory were calculated via linear interpolation of the *u* and *v* wind components. If wind prohibited forward movement along the trajectory, the bird stopped flying until the next time-step. The simulation was run for consecutive migratory start dates (1–10 May) with migration starting at 18.00 UTC, at four different pressure levels, for the years 1979–2007. The starting dates were selected based on field observations of take-off in Mauritania and arrival in France (Dick *et al*. 1987; Piersma *et al*. 1992, 2005). Red knots are considered to predominantly depart for migration in the early evening (Piersma *et al*. 1990; but see Leyrer *et al*. 2009). Corresponding to the weather data time-steps, the model is run in 6 h time-steps; thus 18.00 UTC is the most suitable simulation start time. Each of the above simulations was run for a constant pressure level (see electronic supplementary material for the Matlab simulation code).

Flight times were calculated along the entire trajectory per migratory start date, pressure level and year, resulting in 1160 simulations (10 days, 29 years, 4 pressure levels). The effects of year (*n* = 29), start date (*n* = 10) and pressure level (*n* = 4) were investigated using non-parametric replacements for one-way and two-way analysis of variance, the Kruskal–Wallis test and the Friedman test (Gibbons & Chakraborti 2003), respectively. In our analysis, non-parametric tests are preferred over the normal analysis of variance owing to the skewed distribution of flight times, especially within pressure level. Results are reported as significant for *p*-value < 0.05.

### (b) Red knot counts

Red knots were counted during northward migration at known staging sites at the French Atlantic coastal wetlands during several years between 1979 and 2007 (Leyrer *et al*. in press). Counted numbers were linearly interpolated over the main stopover period (25 April–25 May) for each year and bird-days were calculated from the daily interpolation results (Leyrer *et al*. in press). We expect that in years where winds experienced *en route* are more unfavourable, and hence flight times are longer, more birds will stop in France. The French stopover site is approximately 1000 km from the German Wadden Sea, a distance that can be covered in windless conditions in less than 24 h (four simulation time-steps). We assume that a bird's decision to stop in France is based on the wind experienced so far and an expectation of what would be experienced further along the trajectory. To test whether future wind conditions *en route* can be predicted over France, we calculated the Pearson correlation per pressure level between simulated ground speeds over France and simulated ground speeds further along the trajectory (up to six time-steps), ground speeds being a proxy for wind conditions. The results are provided as auto-correlograms (fig. S1, electronic supplementary material).

Finally, to test the relation between staging site usage in France and wind conditions experienced *en route*, we fitted a linear regression model to the number of birds staging in France (the dependent variable) as a function of the simulated flight time for the entire trajectory, averaged over all the possible departure dates (the independent variable). A separate model was fitted for each pressure level. We calculated the Pearson correlation coefficient to measure the degree of linear association between simulated flight duration and observed bird-days at the French staging site. We provide results for models with significant correlations (*p* < 0.05) only.

## 3. Results

Simulated flight times from Mauritania to the Wadden Sea ranged from 2 to 8 days in total, with the mode at 3.9 days at the 1000 mb pressure level (111 m above mean sea level at standard atmospheric conditions; Anonymous 1976), 3.8 days at 925 mb (766 m a.m.s.l.), 3.1 days at 850 mb (1457 m a.m.s.l) and 2.5 days at 700 mb (3012 m a.m.s.l.; figure 1*b*(i)). Along the migration route, the probability distribution functions of cumulative flight times at each pressure level were positively skewed and the variance was highest for the 700 mb trajectories, especially at the southerly latitudes (figure 1*b*). With strong wind assistance, birds could complete the trajectory within 2 days of non-stop flight, flying at ground speeds of approximately 32 m s^−1^. The mean and median flight times for all pressure levels and start dates were 3.8 and 3.5 days, respectively.

**Figure 2.**
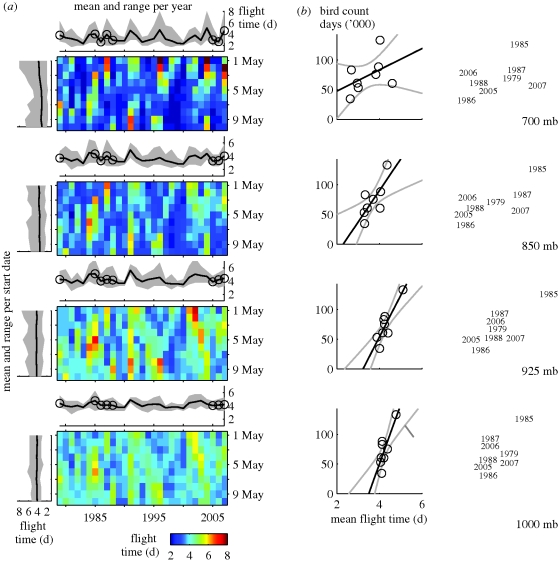
Flight times per simulation and mean flight times in relation to observations. (*a*) The checkerboard plots show simulated number of flight days per pressure level. Each grid cell represents a separate simulation. The graphs in the margins highlight the mean and range of flight time by grouping the simulations per year (above) and per start date (left). The circles in the graphs above the plots represent observations in France. (*b*) Observations (circles) and their corresponding years and linear regression (black line) for number of bird-days and the respective mean flight time per pressure level (*n* = 8). Grey lines represent 95 per cent confidence boundaries.

Measured annual, daily and altitudinal variability in wind strongly affected calculated flight times (figure 2*a*). The distribution of flight times varied significantly between years (Kruksal–Wallis: *H*_28_ = 258.9, *p* < 0.001), without a clear inter-annual pattern. ‘Good years’ (years with very short mean flight times), were characterized by low variability in flight times for different start dates and pressure levels (e.g. 1983, 1986, 1990, 2000 and 2006; figure 2*a*), whereas ‘bad years’ (long mean flight times) were characterized by high variability in flight times. Thus, in ‘good years’ flight times were short regardless of start date and pressure level, whereas in ‘bad years’ flight times could vary significantly depending on start date and pressure level. The distribution of flight times did not differ significantly between start dates (Kruksal–Wallis: *H*_9_ = 5.4, *p* = 0.80) but varied significantly between pressure levels (Kruksal–Wallis: *H*_3_ = 226.5, *p* < 0.001). Wind speed generally increased with altitude. With supporting winds, flight times at 700 mb tended to be shorter than at other pressure levels. However, on occasions with very strong opposing winds, flight times at high altitudes were disproportionately longer than at other pressure levels (figures 1*a*,*b* and 2*a*), on some occasions taking 4 days longer than the flight time at another pressure level (e.g. start date 1 May 2004). When testing the significant factors (pressure level and year) in a two-way model, both factors were also significant (Friedman, pressure level when correcting for year: **χ**^2^_3_ = 290.6, *p* < 0.001; year when correcting for pressure level: **χ**^2^_28_ = 344.9, *p* < 0.001).

To explore the hypothesis of wind-driven stopover site use at the French Atlantic coast, we analysed the linear relation between simulated flight times for the entire trajectory and observed bird numbers on French Atlantic coastal intertidal mudflats. The yearly numbers of bird-days in France were positively and significantly correlated with mean flight times along the trajectory at the 1000 mb pressure level (*r*^2^ = 0.67, *p* = 0.013, *y* = 99.9*x* − 348.7), the 925 mb pressure level (*r*^2^ = 0.76, *p* = 0.005, *y* = 70.0*x* − 225.5) and the 850 mb pressure level (*r*^2^ = 0.55, *p* = 0.035, *y* = 53.1*x* − 121.7; figure 2*b*).

In our model we implicitly assume that a bird's decision to use an emergency stopover site is based on the winds already experienced *en route*, as well as those expected in the near-future time-steps further along the trajectory. Ground speeds over France (a proxy for wind conditions) are positively linearly correlated (*p* < 0.05) with ground speeds 3–5 six-hour simulation steps further along the trajectory (depending on the pressure level; fig. S1, electronic supplementary material). Thus, wind conditions in France are highly auto-correlated in space and time with conditions further along the route, sometimes up to 30 h in advance.

## 4. Discussion

We showed that in years with little wind assistance, resulting in longer flight times and hence increased energy expenditure, many more red knots used the French stopover sites than in years with supporting winds *en route*. Thus, in years with limited wind assistance, the birds make an additional refuelling stop before arriving in the German Wadden Sea. Red knots staging in France were much lighter than any of the birds subsequently found in Germany (Dick *et al*. 1987; Piersma *et al*. 1992; van de Kam *et al*. 2004), providing evidence that they had run out of energy stores and suggesting that they use the French inter-tidal areas as an emergency staging site. In systems like in the Afro-Siberian red knot system, where birds are unable to predict winds *en route* based on conditions at the onset of migration (Piersma *et al*. 1990), they can either deposit extra fuel stores or make strategic use of emergency stopover sites, here provided by the Central French Atlantic coastal wetlands.

Optimal migration theory predicts that birds minimizing migration duration would benefit from skipping sites with lower or unpredictable food supplies; variability in food supply between years or stopover sites could thus explain the skipping seen in some species (Gudmundsson *et al*. 1991; Weber *et al*. 1994; Alerstam & Hedenström 1998; Beekman *et al*. 2002; Bauer *et al*. 2008). Currently, there is no evidence showing that the quality of resources in the French stopover site are lower or more variable than at the wintering site in Mauritania or the primary stopover site in the Wadden Sea (Leyrer *et al*. in preparation). We believe that our results strongly support the hypothesis that wind experienced *en route*, perhaps in combination with the average level of energy stores achieved at the onset of migration, determines the erratic use of intermediate and emergency stopover sites. The emergency stopover strategy is a facultative response to changing environmental conditions and can be seen as an example of an ‘emergency life-history stage’, which can be defined as rapid behavioural and physiological responses to short-term unpredictable events (Wingfield 2003).

Although mean as well as median flight times were shorter at the 700 mb pressure level, there were occasions when strong opposing winds resulted in the longest flight times (8 days). Counts of red knots in France were most strongly correlated with flight times at the 925 mb pressure level. Our findings suggest that flight at lower altitudes might be more reliable (including smaller chances to be blown off course) than at higher altitudes. Our results also show that wind conditions (assessed by comparing ground speeds) in France are highly auto-correlated in space and time up to 18 h in advance and further along the trajectory. This suggests that as birds approach an emergency stopover site such as the French Atlantic coastal wetlands, they can decide whether to use that site based on their current energetic state and their short-term future expectation of wind conditions and energetic expenditure along the trajectory. Spatial explicit simulation modelling facilitates the exploration of such relationships (e.g. Erni *et al*. 2005; Vrugt *et al*. 2007) and would enable researchers to study the extent of such spatio-temporal auto-correlations along an entire trajectory or trajectory segments in contrast to considering only temporal autocorrelation of wind conditions on stopover decisions (e.g. Weber & Hendenström 2000).

Given the ubiquity of stochastically varying media (air and water), due for example to wind and currents, we expect such emergency habitats to exist in many other systems, both airborne and oceanic migration systems. For example, in marine animals emergency site use owing to ocean currents might be reflected in variable visits to foraging areas following the breeding season, such as in adult green turtles *Chelonia mydas*, which cannot forage while crossing the open ocean to reach coastal foraging sites (Godley *et al*. 2002). In the well-studied model system of the red knot, the existence of emergency habitats is observable; however, in other migratory systems (e.g. passerine or insect migration) they may remain unidentified owing to the high variability in their use. Detecting such emergency staging areas in oceanic environments will prove to be exceptionally challenging because of the difficulty in observing marine animal movements (Godley *et al*. 2002; Luschi *et al*. 2003; Rutz & Hays 2009; Sale & Luschi 2009). This has serious implications for conservation measures. Migrating animals depend on the integrity of multiple stopover sites, the importance of which is not always readily seen. If we are to protect migratory species and the migration phenomenon, proactive conservation measures are needed to protect species while they are still abundant (Wilcove & Wikelski 2008) by identifying and protecting habitats along migration routes, even when they are not consistently designated as migratory hotspots. Our model can easily be applied to other migratory systems to quantify the impact of wind on flight times along a migration trajectory and help identify cases where emergency stopover sites might be crucial for migratory success. Similarly, our modelling approach could be applied to marine systems, where currents affect migration as well as foraging movements of marine species (McCleave 1993; Luschi *et al*. 2003; Brooks *et al*. 2009; Sale & Luschi 2009), provided current data are available. We believe that the comparison of simulation models integrating dynamic environmental conditions with field measurements (Shamoun-Baranes *et al*. 2006; Bauer *et al*. 2008; Felicísimo *et al*. 2008) and the concurrent improvement of bio-logging technologies (Cooke *et al*. 2004; Wikelski *et al*. 2006; Rutz & Hays 2009) will greatly enhance migration research (Bauer *et al*. 2009).
